# Distributed Task Offloading in Heterogeneous Vehicular Crowd Sensing

**DOI:** 10.3390/s16071090

**Published:** 2016-07-14

**Authors:** Yazhi Liu, Wendong Wang, Yuekun Ma, Zhigang Yang, Fuxing Yu

**Affiliations:** 1 College of Information Engineering, North China University of Science and Technology, Tangshan 063009, China; mayuekun@163.com (Y.M.); yzg-lzy@163.com (Z.Y.); yfx626@126.com (F.Y.); 2 State Key Laboratory of Networking and Switching Technology, Beijing University of Posts and Telecommunications, Beijing 100876, China; wdwang@bupt.edu.cn

**Keywords:** vehicular crowd sensing, mobile crowd sensing, task offloading

## Abstract

The ability of road vehicles to efficiently execute different sensing tasks varies because of the heterogeneity in their sensing ability and trajectories. Therefore, the data collection sensing task, which requires tempo-spatial sensing data, becomes a serious problem in vehicular sensing systems, particularly those with limited sensing capabilities. A utility-based sensing task decomposition and offloading algorithm is proposed in this paper. The utility function for a task executed by a certain vehicle is built according to the mobility traces and sensing interfaces of the vehicle, as well as the sensing data type and tempo-spatial coverage requirements of the sensing task. Then, the sensing tasks are decomposed and offloaded to neighboring vehicles according to the utilities of the neighboring vehicles to the decomposed sensing tasks. Real trace-driven simulation shows that the proposed task offloading is able to collect much more comprehensive and uniformly distributed sensing data than other algorithms.

## 1. Introduction

In mobile crowd sensing systems, ordinary citizens are recruited to collect and share data from their surrounding environments through mobile devices [1,2]. On the basis of collected sensing data, mobile crowd sensing systems are able to provide users with various novel applications [3], such as real-time air quality reports [4] and road traffic monitoring [5,6]. Various sensing devices and mobile communication interfaces are installed in an increasing number of vehicles, which are able to collect and share various types of sensing data in urban environments. The extensive distribution of these vehicles in urban areas has realized vehicular mobile crowd sensing.

In a vehicular mobile sensing system, sensing data are collected by vehicles and then transmitted to a sensing data center via wireless networks. Vehicular mobile crowd sensing can collect a large amount of comprehensive data, which are collaboratively collected by multiple sensing vehicles. To meet the sensing data requirements of vehicular mobile crowd sensing applications, a large amount of sensing tasks should be generated by the sensing system to collect user-required sensing data. Therefore, vehicular mobile crowd sensing systems mainly collect various kinds of sensing data that encompass all the tempo-spatial dimensions required by sensing tasks. However, sensing vehicles are heterogeneous in terms of sensing interfaces and mobility trajectories. Thus, collecting all required data for sensing tasks by one sensing vehicle is impossible.

Sensing data are temporal, and collected sensing data may expire after certain time. For example, atmospheric temperature changes with time in any given day; as a result, collected temperature data will be expired after a few hours. Moreover, different types of sensing data may have different life cycles. For example, the life cycle of noise data is much shorter than that of temperature data. Thus, vehicular crowd sensing systems should continuously generate sensing tasks and collect sensing data in target sensing areas to ensure relevance of the user-required data.

In vehicular mobile crowd sensing systems, the quality of collected sensing data increases with the increase in number of sensing vehicles used to collect data, which, consequently, equates to high cost [7]. Therefore, continuous collection of tempo-spatial sensing data required for a task with a limited number of heterogeneous sensing vehicles is a critical issue.

This work proposed a utility-based sensing task decomposition and offloading algorithm. A mobility model based on a continuous-time Markov chain is established to forecast the spatial distribution of sensing vehicles. A utility function is designed to estimate the sensing task execution capacity of sensing vehicles on the basis of the spatial distribution and interfaces of sensing vehicles, tempo-spatial coverage of collected data, requirements of the tempo-spatial coverage, and types of sensing data. Then, the sensing tasks are decomposed and offloaded to neighboring vehicles according to the utilities of the neighboring vehicles to the decomposed sensing tasks.

The remainder of this paper is organized as follows. Section 2 reviews the related studies on mobile crowd sensing and vehicle sensing. Section 3 presents the vehicular mobile crowd sensing system model, the tempo-spatial sensing data model, and the mobility model of the sensing vehicles. A sensing utility function of a vehicle to sensing tasks is proposed in Section 4, based on the heterogeneity of the sensing abilities of the sensing vehicles and the sensing task required sensing data tempo-spatial coverage. Then, a sensing Task Offloading algorithm is proposed based on the proposed utility function in Section 4. Section 5 evaluates the performance of the proposed strategy through real trace-driven simulations. Section 6 presents the conclusions.

## 2. Related Work

Mobile crowd sensing [1] is a promising means of collecting comprehensive sensing data in urban areas. Different from traditional wireless sensor networks [8,9], sensors are equipped in mobile phones or on board units and carried by people or vehicles. In recent years, a large number of novel applications [3] based on mobile crowd sensing have been deployed. For example, The Common Sense project [10] monitored the contents of pollutants in the air by collecting sensing data from handheld air quality monitors. Yu et al. [11] recovered noise situation throughout New York City (NYC) based on mobile crowd sensing and inferred the fine-grained noise situation at different times of the day for each region of NYC with the use of data from the city complaint platform, together with social media, road network data, and points of interests. The Mahali project [12] used GPS signals that penetrate the ionosphere for science rather than positioning; a large number of ground-based sensors fed data into a cloud-based processing environment through mobile devices, thus enabling a tomographic analysis of the global ionosphere at an unprecedented resolution and coverage. Zhou et al. [13] presented a novel bus arrival time prediction system based on crowd sensing and predicted bus arrival times by encouraging passengers to collect generally available and energy-efficient sensing resources, including cell tower signals, movement statuses, and audio recordings. The TomTom City project [14] provides live and historical traffic and travel information services, and the traffic data is collected by mobile vehicles. However, existing mobile sensing applications suffer from poor sensing data qualities owing to the lack of theoretical research on mobile crowd sensing; thus, the commercial deployment of mobile crowd sensing systems is restricted.

Zhang et al. [15] proposed a four-stage life cycle (i.e., task creation, task assignment, individual task execution, and crowd data integration) to characterize the mobile crowd sensing process. Selecting the most efficient sensing nodes to collect sensing data is a commonly used method in task creation and assignment stages to guarantee coverage of sensing data [16]. The basic principle of participant selection is to collect sensing data with the highest coverage ratio by limited number of participants [17]. Reddy et al. [18] developed a selection framework to allow organizers to identify well-suited sensing nodes for data collection on the basis of their geographic and temporal availabilities, as well as their habits. Tuncay et al. [19] exploited the stability of user behavior and selected sensing nodes according to the fitness of the mobility history profiles of users. Moreover, modeling the mobility of sensing nodes and forecasting the traces of sensing nodes can reduce the required amount of participating sensing nodes [20,21]. When selecting sensing nodes, forecasting the traces of the nodes with their call logs can reduce energy consumption and result in high sensing data coverage ratio [22].

Song et al. [23] satisfied most requirements of sensing tasks by limited incentives to achieve favorable coverage ratio of collected sensing data. Guang et al. [24] constructed contractual arrangements as incentive mechanism for system administrator to encourage participants to help detecting the malicious user. Chien et al. [25] introduced the online task assignment problem, in which heterogeneous tasks are assigned to workers with different unknown skill sets. Furthermore, this method is able to complete more sensing tasks in time because both the target location of the sensing tasks and the movement range of the participants are considered [26].

Frequent encounters between mobile vehicles provides sensing vehicles opportunity to collaborate with each other. Xiang et al. [27] reduced energy consumption of mobile phone sensing by designing a cloud-assisted collaborative sensing method. Bin et al. [28] generated context characteristics according to collaborative sensing. MOSDEN (mobile sensor data engine) [29] operated on smartphones to collect and share sensing data among multiple distributed applications and users.

Collaboration between vehicles in the task execution stage can further improve the coverage of collected sensing data. For example, in the task execution stage, offloading sensing tasks from mobile devices to the environmental embedded sensors can improve quality of collected sensing data and reduce energy consumption of mobile devices [30,31]. In mobile cloud computing, task offloading [32] migrates the heavy computation task from mobile devices to the cloud to reduce load of mobile devices [33]. Mtibaa et al. [34] proposed a mobile device cloud framework, in which the computing task could be offloaded from one device to another upon interaction. Zheng et al. [35] proposed a participatory computing paradigm to harness the computational power on mobile devices. They propose a general randomized task assignment frame work to minimal workload for individual participating devices. In mobile crowd sensing, Ma et al. [36] delivered collected sensing data between mobile devices to improve data transmission efficiency. Thus, we introduce sensing Task Offloading into the task execution stage to improve quality of collected sensing data.

In early vehicle-based environmental sensing applications, vehicles were used to monitor locations [37], air quality [38], rainy weather [39], or a street view map [40]. However, sensing data are not shared among vehicles in these applications. On the other hand, the emerging Vehicular Ad Hoc Networks (VANETs) reduces the cellular traffic for in-vehicle data services in a cost effective way [41]. Therefore, vehicles are able to exchange information with each other by VANETs [42]. In a previous study [43], multiple probe vehicles were utilized to estimate large-scale traffic in an urban environment. Our previous work [44] proposed a collaborative sensing strategy to collect top quality sensing data with limited sensing vehicles. Vehicular collaborative sensing can also be utilized for safe driving [45]. Collecting all sensing data required by applications is the foremost criterion of vehicular mobile crowd sensing systems. However, none of the previous studies considered decomposition and offloading sensing tasks between sensing vehicles to improve efficiency of sensing task execution.

## 3. System Model

### 3.1. System Architecture

**Figure 1:**
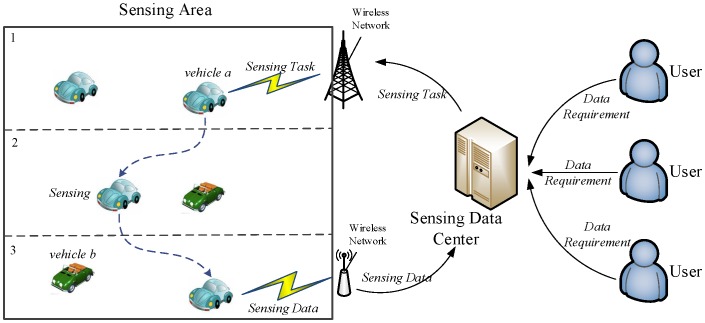
Architecture of vehicular mobile crowd sensing systems.

A vehicular mobile crowd sensing system is composed of sensing data servers, wireless networks, and vehicles (i.e., sensing nodes), as shown in Figure 1. A sensing node is a vehicle installed with multiple types of sensing devices and wireless communication interfaces. All sensing nodes should be willing to participate in the collection of sensing data. The sensing data center is in charge of a vehicular sensing system, and collected sensing data are stored in the sensing data center. Users of the sensing system can make queries on the sensing data of interest from the data server. Then, the data requirements are delivered to sensing vehicles in the form of sensing tasks.

**Figure 2:**
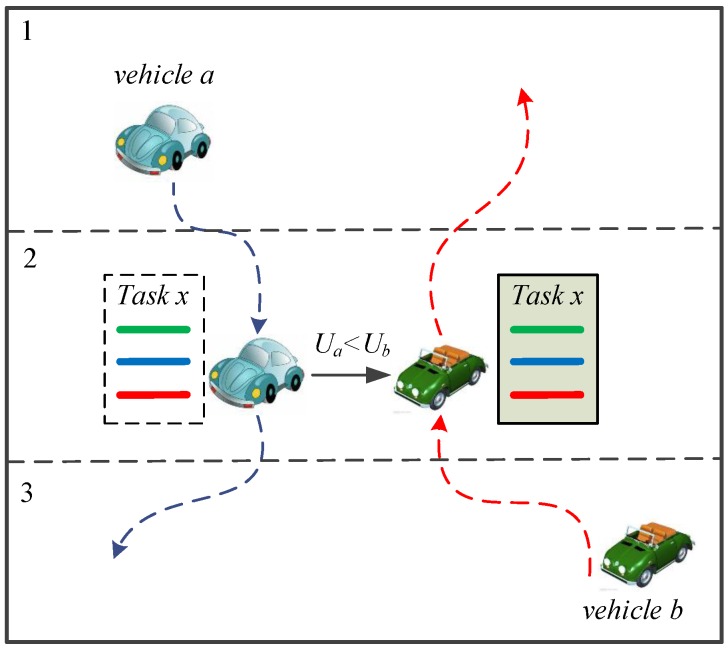
Sensing task offloading.

The sensing data center generates sensing tasks according to the data requirements from the system users. Then, the sensing tasks are delivered to the sensing vehicles to collect matching sensing data. Sensing task-based data collection can collect data with limited sensing resources. In practical scenarios with a significantly large number of sensing tasks and available heterogeneous sensing vehicles, the execution of a sensing task by an appropriate sensing vehicle becomes an arduous problem. This work presents a sensing task decomposition and offloading strategy (Figure 2) that can execute as many as possible sensing tasks with a limited number of vehicles.

### 3.2. Sensing Data Model

The geographic area where the vehicular crowd sensing system is going to collect sensing data is called target sensing area (or sensing area). The sensing area is divided into lattice cells based on the geographical location (Figure 2), and each lattice cell is called a subarea. A subarea is a unit area of the sensing system; the assumption is that sensing data sampled at any point in the subarea can indicate the sensing value of the subarea. The entire sensing area is divided into *N* subareas in the form of grids based on geographical coordinates (Figure 2). Let $I=\left\{1,2,\cdots ,N\right\}$ denote the set of subareas in the sensing area. Let *i*(i∈I,1⩽i⩽N) denote one of the subareas. Table 1 lists notations used in this paper.

Given that sensing data are usually sensitive to time (e.g., air temperature data collected an hour ago are no longer accurate), sensing data should be sampled periodically in each unit sensing area. We suppose that there are *M* types of sensing interfaces embedded in the vehicle sensing nodes. Let $w_{l}$ denote the validity period of the *l*th type of sensing data. Let $Y_{\alpha }=\left(y_{1}^{\alpha },y_{2}^{\alpha },\cdots ,y_{M}^{\alpha }\right)$ denote the sensing ability of the vehicular sensing node *α*,

(1) $$y_{l}^{\alpha }=\left\{\begin{array}{c} 0\\ 1 \end{array}\right.$$

where $y_{l}^{\alpha }=1$ indicated that *α* can collect the sensing data of type *l*, and $y_{l}^{\alpha }=0$ indicates that *α* cannot collect the data of type *l*.

Vehicles collect sensing data of a specific subarea when it is travelling in the subarea. Let $\xi_{i}^{\alpha }$ denote the moment when vehicle *α* travels into subarea *i*, and $\theta_{i}^{\alpha }$ denote the duration vehicle *α* stay in subarea *i*. Then, the valid function of the sensing data of type *l* collected by *α* when it travels in subarea *i* could be denoted as follows:

(2) $$h_{\alpha }\left(i,l,t\right)=\left\{\begin{array}{cc} y_{l}^{\alpha } & \xi_{i}^{\alpha }⩽t⩽\xi_{i}^{\alpha }+\theta_{i}^{\alpha }+w_{l}\\ 0 & other \end{array}\right.$$

$Z\left(t\right)$ is the valid sensing data in the sensing data center at time *t*.

(3) $$Z\left(t\right)=\left[\begin{array}{cccc} \zeta_{1,1}\left(t\right) & \zeta_{1,2}\left(t\right) & \cdots & \zeta_{1,M}\left(t\right)\\ \zeta_{2,1}\left(t\right) & \zeta_{2,2}\left(t\right) & & \zeta_{2,M}\left(t\right)\\ \vdots & & & \vdots \\ \zeta_{N,1} & \zeta_{N,2}\left(t\right) & \cdots & \zeta_{N,M}\left(t\right) \end{array}\right]$$

where $\zeta_{i,l}\left(t\right)$ is the valid function of the sensing data of type *l* in subarea *i* at time *t*.

(4) $$\zeta_{i,l}\left(t\right)\left\{\begin{array}{cc} 1 & \text{the}\;\text{sensing}\;\text{data}\;\text{is}\;\text{effective}\;\text{at}\;t\\ 0 & \text{the}\;\text{sensing}\;\text{data}\;\text{is}\;\text{ineffective}\;\text{at}\;t \end{array}\right.$$

### 3.3. Mobility Model

In the vehicular crowd sensing system, vehicles move in and transfer between the subareas. Let $X=\left\{X\left(t\right),t⩾0\right\}$ denote the mobility procedure, where X(t) is the state of the vehicle at time *t* (i.e., the subarea in which the vehicle stays at time *t*). Normally, the state of the vehicle is only related to its state at the last moment. Thus, for any $0⩽t_{0}<t_{1}<\cdots <t_{n}<t_{n+1}$, and $i_{k}\in I$, 0⩽k⩽n+1, we have

(5) $$\begin{array}{c} P\left\{X\left(t_{n+1}\right)=i_{n+1}|X\left(t_{0}\right)=i_{0},X\left(t_{1}\right)=i_{1},\cdots ,X\left(t_{n}\right)=i_{n}\right\}\\ =P\left\{X\left(t_{n+1}\right)=i_{n+1}|X\left(t_{n}\right)=i_{n}\right\} \end{array}$$

Equation (5) indicates that the vehicle mobility procedure $X=\left\{X\left(t\right),t⩾0\right\}$ is a time-continuous Markov chain. For any s,t⩾0, and i,j∈I, we have

(6) $$\begin{array}{c} P\left\{X\left(s+t\right)=j|X\left(s\right)=i\right\}\\ =P\left\{X\left(t\right)=j|X\left(0\right)=i\right\}\\ =P_{ij}\left(t\right) \end{array}$$

where $P_{ij}\left(t\right)$ is the transition probability of a vehicle from subarea *i* to subarea *j* after time duration *t*. Let $P\left(t\right)=\left(P_{ij}\left(t\right)\right)$, i,j∈I denote the matrix of transition probability.

We define the transfer rate matrix of the mobility procedure $X=\left\{X\left(t\right),t⩾0\right\}$ as $Q=\left(q_{ij}\right)$ , which is also called the *Q* matrix. For any i∈I, we define

(7) $$q_{ii}=\lim_{\Delta t\to 0}\frac{1-P_{ii}\left(\Delta t\right)}{\Delta t}$$

For any i,j∈I, j≠i

(8) $$q_{ij}=\lim_{\Delta t\to 0}\frac{P_{ij}\left(\Delta t\right)}{\Delta t}$$

where $q_{ij}$ is the transition intensity of the vehicle from subarea *i* to subarea *j*. Since the number of the subareas in the sensing area is limited, for ∀i∈I, $0<\sum_{j\neq i}q_{ij}=q_{i}<\infty$. The average length of the duration that the vehicle stay in state $X\left(0\right)=i$ is determined by $q_{i}$. Thus, the value of $q_{i}$ can be estimated by the historical mobility traces of the vehicles.

According to the Kolmogorov backward differential equation

(9) $$P^{\prime }\left(t\right)=\mathrm{QP}\left(t\right)$$

Let $P\left(0\right)=I$. Then, the transition probability of the Markov chain *X* could be derived as follows:

(10) $$P\left(t\right)=e^{Qt}$$

## 4. Sensing Task Offloading

In this section, a utility function is designed to estimate the sensing task execution capacity of sensing vehicles on the basis of the spatial distribution and interfaces of sensing vehicles, tempo-spatial coverage of collected data, requirements of the tempo-spatial coverage, and types of sensing data. Then, the sensing tasks are decomposed and offloaded to neighboring vehicles according to the utilities of the neighboring vehicles to the decomposed sensing tasks. By decomposing and offloading the sensing tasks, each part of the sensing tasks can be executed more efficiently. Therefore, the sensing efficiency of the whole sensing system will be improved.

### 4.1. Sensing Task Decomposition

Let $T_{k}=\left\{I_{k},l_{k},{\tilde{t}}_{k}\right\}$ denote a sensing task, where $I_{k}$ is a set of target subareas of the sensing task, $l_{k}$ is the required sensing data types of the sensing task, and ${\tilde{t}}_{k}$ is the temporal coverage duration of required sensing data. We define the utility of sensing vehicle *α* to task $T_{k}$ as follows:

(11) $$u_{\alpha }\left(T_{k}\right)=\sum_{i\in I_{k}}^{}\sum_{l\in l_{k}}^{}\int_{t\in {\tilde{t}}_{k}}u_{\alpha }\left(i,l,t\right)dt$$

where $u_{\alpha }\left(i,l,t\right)$ is the utility of sensing vehicle *α* to the sensing data of type *l* in subarea *i* at time *t*.

(12) $$u_{\alpha }\left(i,l,t\right)=h_{\alpha }\left(i,l,t\right)-h_{\alpha }\left(i,l,t\right)\zeta \left(i,l,t\right)$$

where $h_{\alpha }\left(i,l,t\right)$ is the effective duration length of the sensing data of type *l* collected by *α* in subarea *i*. The calculation of the utility of a node will consume resources of the sensing node, and the node should predict its location in finite time. Thus, a system duty cycle $t_{u}$ is set to reduce the cost of utility calculation. The vehicle trace prediction is within one system duty cycle $t_{u}$. The utility of the vehicle sensing nodes are also updated on a $t_{u}$ cycle. If *α* arrives the subarea *i* at time *t*, and then stay in the subarea *i* for time *τ*, (i.e., $\xi_{i}^{\alpha }=t$, $\theta_{i}^{\alpha }=\tau$), then

(13) $$\int_{t=0}^{t_{u}}h_{\alpha }\left(i,l,t\right)dt=\tau +w_{l}$$

Thus, for any $\xi_{i}^{\alpha }$ and $\theta_{i}^{\alpha }$, we have

(14) $$\begin{array}{c} \int_{t=0}^{t_{u}}h_{\alpha }\left(i,l,t\right)dt\\ ={\;\int \int \;}_{t\in \left(0,t_{u}\right),\tau \in \left(0,t_{u}-t\right)}\left\{P\left(\xi_{i}^{\alpha }=t\right)\cdot P\left(\theta_{i}^{\alpha }=\tau \right)\cdot \left(\tau +w_{l}\right)\right\}\cdot d\tau dt \end{array}$$

where $P\left(\xi_{i}^{\alpha }=t\right)$ is the probability density of the time that vehicle *α* arrives at subarea *i*. $P\left(\theta_{i}^{\alpha }=\tau \right)$ is the probability density of the duration that vehicle *α* stays in subarea *i*.

The sensing task $T_{k}$ is decomposed into *n* subtasks $T_{k1}\cdots T_{kn}$ based on the expressions in Equations (15)–(17)

(15) $$\overset{n}{\underset{\alpha \;=\;1}{⋂}}T_{k\alpha }=\emptyset$$

(16) $$\overset{n}{\underset{\alpha =1}{⋃}}T_{k\alpha }=T_{k}$$

(17) $$u_{\alpha }\left(i,l,t\right)⩾u_{\beta }\left(i,l,t\right),\forall \left(i,l,t\right)\in T_{k\alpha },\alpha ,\beta \in \Omega .$$

Equations (15) and (16) are the restrictions to sensing task decomposition. It means that the decomposed tasks are not overlapped and the union of all the subtasks equals to the origin sensing task. Equation (17) decomposes the sensing task according to the utility function of the neighbor nodes to the sensing task.

### 4.2. Distribution of Residence Time

If the sensing node arrives in subarea *i* at time 0, then $X\left(0\right)=i$. If $\theta_{i}$ is the residence time of a vehicle in subarea *i*, the residence time $\theta_{i}$ can be defined as

(18) $$\theta_{i}=\inf\left\{t:t>0,X\left(t\right)\neq X\left(0\right),X\left(0\right)=i\right\}$$

then

(19) $$P\left(\theta_{i}>t|X\left(0\right)=i\right)=P\left[X\left(u\right)=i,0⩽u⩽t|X\left(0\right)=i\right]$$

First, the uncountable events are translated into countable events, as shown in Equation (20).

(20) $$B=\left\{\Upsilon :X\left(u\right)=i,0⩽u⩽t\right\}=\underset{0⩽u⩽t}{⋂}\left\{\Upsilon :X\left(u\right)=i\right\}$$

where *B* indicates that the sensing node stays in subarea *i* in time duration [0,t]. Then, the interval [0,t] is uniformly divided into $2^{n}$ subintervals as follows:

(21) $$\begin{array}{c} A_{n}=\left\{\Upsilon :X\left(\frac{k}{2^{n}}t\right)=i,k=0,1,2,\cdots ,2^{n}\right\}\\ =\overset{2^{n}}{\underset{k=0}{⋂}}\left\{\Upsilon :X\left(\frac{k}{2^{n}}t\right)=i\right\}. \end{array}$$

$A_{n}$ is the event where the sensing node is in subarea *i* at time $\frac{k}{2^{n}}t$, where $k=0,1,2,\cdots ,2^{n}$. Given that: $A_{n+1}\subset A_{n}$, it could be denoted as follows:

(22) $$A=\overset{\infty }{\underset{n=1}{⋂}}A_{n}=\lim_{n\to \infty }A_{n}$$

*A* also indicates the event where the sensing node stays in subarea *i* from time 0 to time *t*. Obviously, B=A. Therefore,

(23) Pθi>t|X0=i=limn→∞PAn|X0=i=limn→∞exp2nlnPiit2n=limn→∞expln1-qit2n+ot2n-qit-qit2n2n-qit=exp-qit

Thus, the residence time of a vehicle in subarea *i* is subject to the exponential distribution with parameter $q_{i}$. The average residence time in the subarea is denoted as $\lambda_{i}$, and $\lambda_{i}=q_{i}$, which means that the parameter of the exponential distribution can be estimated by the average residence time of the sensing vehicles in the subarea.

Consequently, $\theta_{i}$ obeys the exponential distribution with parameter $\lambda_{i}$, which is the average residence time of the vehicle in subarea *i*.

(24) $$P\left\{\theta_{i}⩽t|X\left(0\right)=i\right\}=1-\exp\left(-q_{i}t\right)$$

where $q_{i}=\lambda_{i}$.

### 4.3. Distribution of Arrival Time

The arrival time of a vehicle in subarea *j* from subarea *i* (i.e., t = 0) is denoted as $\xi_{ij}$ and defined as

(25) $$\xi_{ij}=\inf\left\{t:t>0,X\left(t\right)=j|X\left(0\right)=i\right\}$$

where $\xi_{ij}=0$ if $X\left(0\right)=j$.

$F_{ij}\left(t\right)$ is defined as the distribution function of $\xi_{ij}$ and is expressed as

(26) $$F_{ij}\left(t\right)=P\left\{\xi_{ij}⩽t\right\},i,j\in I,i\neq j,t⩾0.$$

If $\vartheta_{1}=\inf\left\{t:t>0,X\left(t\right)\neq X\left(0\right)\right\}$ is the moment that the vehicle leaves its initial state and

(27) $$G_{i}\left(x\right)=P\left(\vartheta_{1}⩽x|X\left(0\right)=i\right)=\left(1-\exp\left(-q_{i}x\right)\right)$$

then

(28) $$F_{ij}\left(t\right)=q_{i}^{-1}q_{ij}G_{i}\left(t\right)+q_{i}^{-1}\sum_{k\neq i,k\neq j,k\in I}q_{ik}\int_{0}^{t}F_{kj}\left(t-u\right)dG_{i}\left(u\right)$$

If $\vartheta^{\prime }=\inf\left\{t:t>0,X\left(\vartheta_{1}+t\right)=j\right\}$ and $X^{\prime }\left(t\right)=X\left(\vartheta_{1}+t\right)$, then $\vartheta =\vartheta_{1}+\vartheta^{\prime }$. Based on the total probability formula and the strong Markov property, $\vartheta_{1}$ and $X\left(\vartheta_{1}\right)$ are conditionally independent when $X\left(0\right)=i$; thus, Equation (28) is true.

### 4.4. Distributed Task Offloading

Utility of a sensing vehicle to a sensing task can be derived based on the historical traces of the vehicles, sensing interfaces of the participant vehicles, and task requirements in terms of sensing data types and tempo-spatial coverage. We divide time into duty for convenient calculation. Therefore, each task consists of unit tasks, and each unit sensing task covers a unit subarea, sensing data type, and unit duty cycle.

In Algorithm 1, each utility of the neighbor nodes to each unit sensing task is firstly calculated. Then, the unit tasks are assigned to different neighbors according to the utilities of the neighbors to the unit tasks. We suppose the task set of sensing vehicle *α* is T; the Task Offloading algorithm is shown as Algorithm 1. In Algorithm 1, T is the task set of *α*. $T_{k}\in T$ is a task in T. Firstly, *α* identify its neighbor set N. Then, each $T_{k}\in T$ is divided into unit tasks $\left\{T_{u}\right\}$. For each $T_{u}$, find the neighbor *β* who has the highest efficiency to execute unit task $T_{u}$. Then, insert $T_{u}$ into task set $T_{\beta }$, which will be offloaded to *β* after all tasks $T_{k}\in T$ is processed.

**Algorithm 1:** Distributed Sensing Task Offloading Algorithm
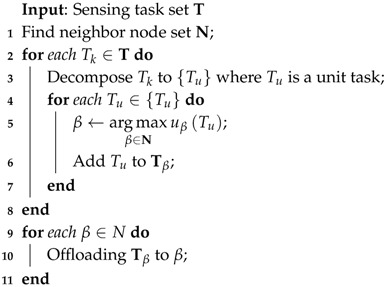


## 5. Experimental Section

T-Drive trajectory [46,47] is utilized to simulate and evaluate the proposed Task Offloading strategy. The T-Drive trajectory includes one-week trajectories of 10357 taxis. The total number of GPS points in this data set is approximately 15 million, and the total distance of the trajectories is 9 million km. The time frequency of location data sampling in T-Drive is set to 5 s. Taxi drivers can usually find an optimal route to a destination based on their experience. Therefore, the T-Drive project attempts to improve the efficiency of the navigation software according to the experience of taxi drivers. The traces of taxis can reflect the characters of passengers. The mobility of taxis is also predictable given that the mobility of passengers is not completely random. For example, Huang et al. [48] designed a vehicle mobility model on the basis of the regular patterns derived from traces of 4000 taxis in Shanghai.

Based on the vehicular traces, we constructed our simulation program using Python language and Numpy package. In the program, we designed a trace based mobility module, where the traces of the 1000 vehicle candidates are preloaded into the memory. Before collecting sensing data and sensing task offloading between sensing vehicles, the vehicles train their time continuous Markov process based mobility model for 24 h. After mobility model training, sensing vehicles periodically collect sensing data and offload sensing tasks according to Algorithm 1.

In our simulations, a rectangular region around the Fourth Ring Road of Beijing is adopted as the target sensing area, between $39.840018^{\circ }$ N and $39.993971^{\circ }$ N, and between $116.276206^{\circ }$ E and $116.494243^{\circ }$ E. The traces in the rectangular area are used in the simulations. Although the traces are not limited in the target sensing area, we abstract the area out of the target sensing area as subarea 0, and the subareas in the target sensing area as 1,2,3,⋯. The 1000 vehicles with the longest traces in the rectangular region are employed as sensing vehicle candidates, and the traces are used to calculate the arrival and residence time distribution among the subareas.

The default parameters of the experiments are set as follows. The default number of participant sensing vehicles is 60, which are selected randomly from the 1000 sensing vehicle candidates. The total time of the experiment is 533,315 s (>6 days). In reality, the value of most types of sensing data can be modeled as a continuous tempo-spatial functions (e.g., temperature or humidity). According to the function, the best sampling tempo-spatial resolution could be estimated based on the Nyquist theorem. In our simulation, the sensing area is a part of the downtown area of Beijing. The rectangular region is uniformly divided into 100 (10×10) subareas. This resolution is supposed to be high enough for the sensing system. The system duty cycle is set to 1000 s. Ten types of sensors are employed to collect data for the sensing system, and each vehicle has 50% possibility to be equipped with each sensor. The default upper bound of each type of sensing data life cycle is 10 system duty cycles. The simulation parameters and their default values are shown in Table 2.

We configured number of sensing vehicles, mean of task durations and task generation rate in our simulation, where number of sensing vehicles ranges from 5 to 100; mean of task duration ranges from 1 system duty cycle to 19 system duty cycle; task generation rate ranges from 10 per system duty cycle to 100 per system duty cycle. We did not configure the mobility model parameters during the simulation procedure because of the mobility of the vehicles is determined by the real traces, which are not configurable.

We compared the Task Offloading algorithm with two other algorithms, namely, Random and Dynamic Participant Selection (DPS) [23]. In the Random algorithm, participatory sensing vehicles are selected randomly from all the participants at the beginning of the simulation. In the DPS algorithm, sensing vehicles are selected according to the utilities of candidate sensing vehicles at the beginning of the simulation.

### 5.1. Results

#### 5.1.1. Impact of the Number of Selected Vehicles

**Figure 3:**
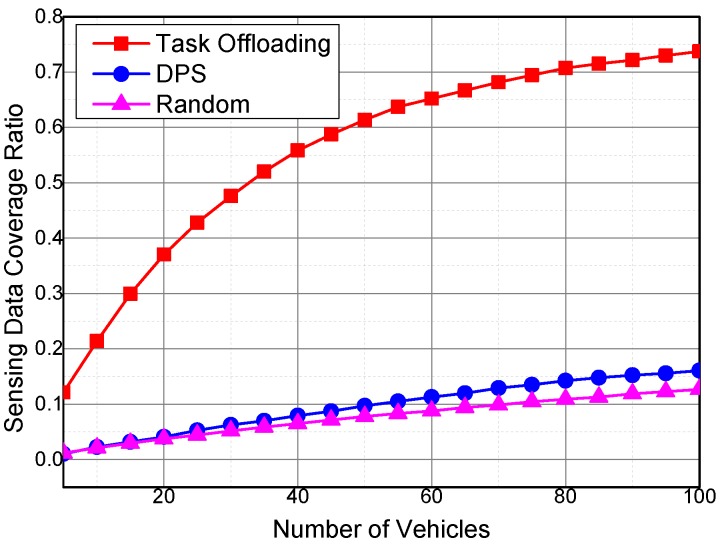
Impact of the number of vehicles on the sensing data coverage ratio.

Figure 3 shows the relationship between the number of vehicles that participated in the sensing data collection and the sensing data coverage ratio. The sensing data coverage ratio $r_{s}$ is the ratio between the temporal coverage of collected sensing data and the product of simulation duration, amount of subareas and number of sensing data types, as shown in Equation (29):

(29) rs=CsCsN×M×TtotalN×M×Ttotal

where $C_{s}$ is cumulative temporal coverage of collected sensing data, *N* is the number of subareas, *M* is the number of sensing data types, and $T_{total}$ is the duration of simulation.

A sensing record is generated when sampling data, which include sensing time, area, data type, and value, of a sensing node are recorded. Furthermore, the length of the validity period of sensing data is determined by the type of sensing data. Therefore, the validity period is also determined after the sensing time is recorded.

We define a 2-D array *S* to count the effective coverage of the collected sensing data. S[i,j] is the effective coverage of sensing data with type *j* in subarea *i*. S[i,j] is a list of ordered time pairs $\left(t_{s},t_{e}\right)$, where $t_{s}<t_{e}$, which denotes a continuous time period. For $\forall p_{x},p_{y}\in S\left[i,j\right]$, if x < y, then $t_{e}$ of $p_{s}$ should be smaller than $t_{s}$ of $p_{y}$. In calculating the cumulative sensing data coverage, we iterate all the collected sensing records to insert effective coverage period pairs into *S*. When inserting one sensing record into *S*, its effective sensing period pair is inserted into *S* according to the type of the sensing data and the subarea where it was collected. The cumulative coverage of collected sensing data should get rid of the impact of overlapping coverage to the same time, subarea and data type. The algorithm for inserting an effective sensing data is shown as Algorithm 2. The input *L* in Algorithm 2 is a list of ordered time pairs and the input *p* is the ordered time pair to be inserted into *L*.

**Figure 4:**
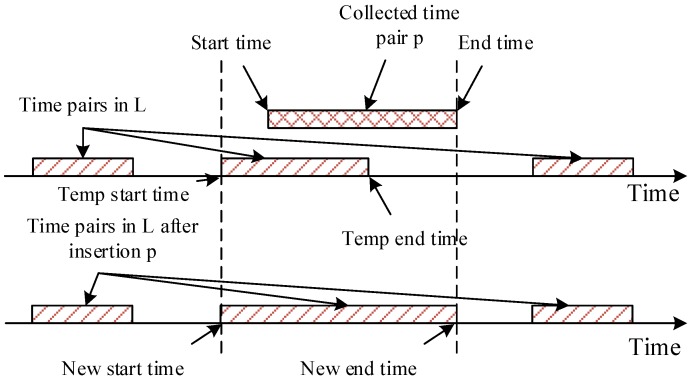
An illustrative diagram to time pair insertion.

In time pair list *L*, time pairs are sequential and non-overlapping as shown in Figure 4. After a sensing data are collected, its temporal coverage is denoted as a time pair. Algorithm 2 inserts the collected time pair *p* into time pair list *L*, as shown in Figure Algorithm 2. In line 20 of Algorithm 2, a target temp time pair $p^{\prime }$ is found in *L*. The start time of *p* is between the end time of $p^{\prime }$ and its precursor in *L*. If the end time of *p* is smaller than the end time of $p^{\prime }$, *p* will not be processed. If the start time of *p* is earlier than the start time of $p^{\prime }$, then the new start time is set to the start time of *p*, else the new start time will be set to the start time of $p^{\prime }$ (line 26–31). Next, the algorithm find the last target pair $p^{\prime \prime }$ who is overlapping with *p* (line 32–42). The new end time is set to the relatively late one in the end time of *p* and $p^{\prime \prime }$ (line 43–47). At last, the new time pair is inserted into *L*, and the covered temp time pairs are deleted from *L* (line 50–57).

Then, the cumulative sensing data coverage $C_{s}$ can be calculated as the sum of all the period lengths in *S*.

In Figure 3, the sensing data coverage ratios of the three algorithms increase with the increase in the number of participating vehicles. The sensing data coverage ratio of the proposed Task Offloading scheme is approximately four times higher than those of the other two schemes. With task partition and offloading, the tasks are offloaded to the vehicles with higher task utilities, and the vehicles with higher utilities are more likely to complete the corresponding task. In the proposed Task Offloading scheme, the heterogeneous sensing data types and temporal cover ages are considered, and one sampling of the sensing data may cover more than one system duty cycle. Both the Random and DPS algorithms do not consider the sensing data lifetime, and each sampling of the sensing data covers only one system duty cycle.

**Algorithm 2:** Time Pair Insertion Algorithm
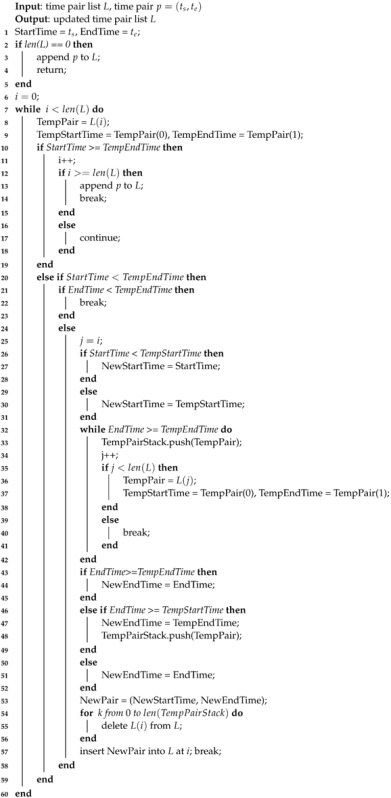


Furthermore, the slope of the Task Offloading curve is also steeper than the other two algorithms. In other words, the sensing data coverage ratio in the Task Offloading algorithm increases faster than that of other two algorithms. If more vehicles are recruited into the system, then more opportunities for collaboration between sensing vehicles are generated, resulting in more chances of offloading one sensing task to a sensing vehicle with higher sensing utility. As a result, the sensing data coverage ratio increases much faster than the other two algorithms.

**Figure 5:**
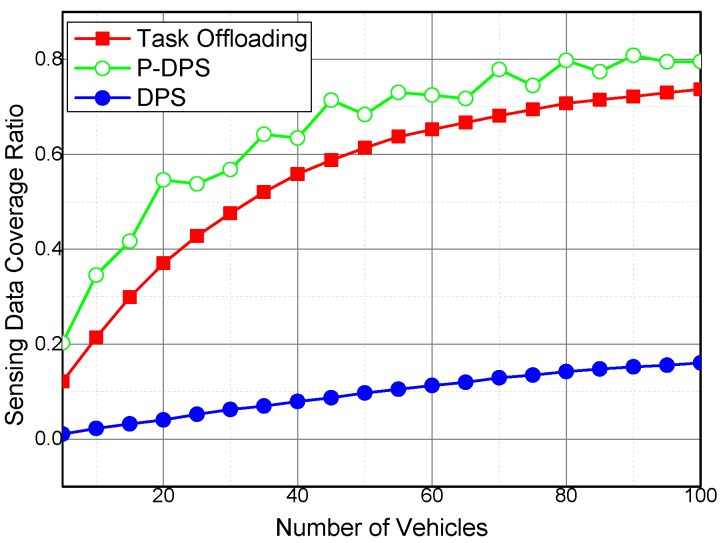
Sensing data coverage ratio of P-DPS, DPS, and Task Offloading.

We also compared the sensing data coverage ratio of the Task Offloading algorithm and DPS with period selection of sensing participants (P-DPS). The duty cycle of the both algorithms is set to 1000 s. For convenience, we take temporal coverage of the sensing data into consideration in P-DPS, and the coverage ratios are shown in Figure 5.

The sensing server can get the utilities of all the sensing vehicles given that a centralized participant selection method is used in the P-DPS algorithm. Then, the P-DPS algorithm is able to select the best participants out of all the candidates to collect sensing data in the following duty cycle. However, P-DPS cannot maintain high performance in a distributed sensing scenario, due to its requirements to global node information. Task Offloading exchanges sensing task between neighboring vehicles in a distributed way to improve quality of collected sensing data. In the proposed Task Offloading algorithm, the location prediction and task decomposition and offloading are utilized to improve the quality of the collected sensing data. Although the sensing data coverage ratio of Task Offloading is lower than that of P-DPS, the performance of Task Offloading is better than that of DPS in the distributed scenarios.

**Figure 6:**
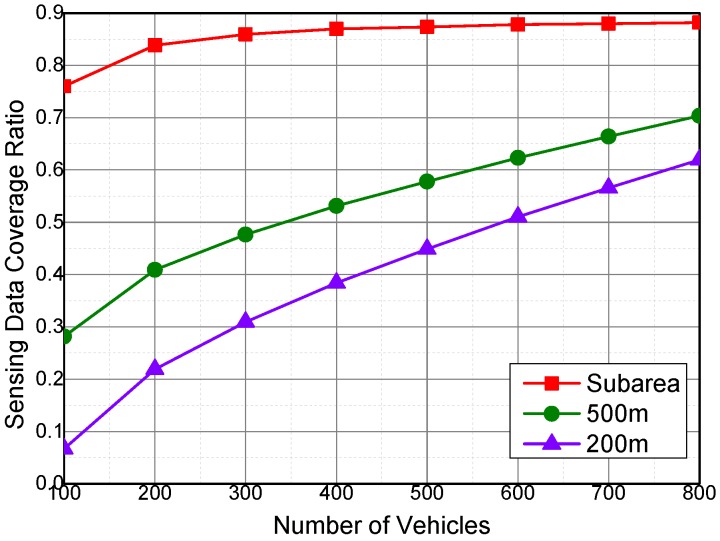
Sensing data coverage ratio with more sensing vehicles.

Figure 6 shows the impact of sensing vehicle amount to the sensing data coverage ratio when the number of sensing vehicles are more than 100. In the first experiment, we suppose that vehicles in a same subarea are neighbors, and they can offload tasks to each other. In the other two experiments, the communication range of the sensing vehicles are set to 500 m and 200 m. Vehicles are neighbors if they are within the communication range of each other. As shown in Figure 6, the coverage ratio raises with the increase of the number of sensing vehicles. But, the increase rate of the coverage ratio decreases along with the number of sensing vehicles. On the other hand, if the communication range is small, the opportunity to offload sensing tasks between the vehicles will be low. As a result, the coverage ratio decreases with the communication range of sensing vehicles.

**Figure 7:**
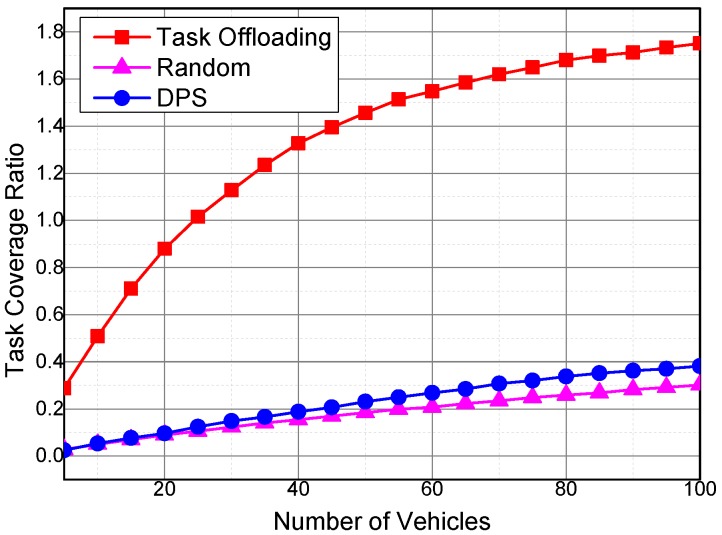
Impact of the number of vehicles on the task coverage ratio.

Figure 7 shows the impact of the number of vehicles on the task coverage ratio. The task coverage ratio $r_{t}$ is calculated as Equation (30),

(30) rt=CsCsCtCt

where $C_{s}$ is cumulative sensing data coverage, and $C_{t}$ is cumulative task requirements. Both $C_{s}$ and $C_{t}$ can be calculated according to Algorithm 2.

The task coverage ratios of the three algorithms exhibit the same variation trend with the sensing coverage ratio under the impact of the number of vehicles. However, the task coverage ratios are higher than that of the sensing data coverage ratios with the same number of sensing vehicles. In our task offloading algorithm, since sensing tasks are decomposed and offloaded, each part of the sensing tasks is offloaded to the most efficient sensing vehicle. As a result, the task offloading algorithm outperforms DPS and Random algorithm, where the sensing tasks are not decomposed and offloaded.

The task coverage ratio is higher than 1 when there are more than 25 sensing vehicles in the Task Offloading algorithm. This phenomenon is unlikely because the task coverage ratio is the ratio between the coverage of collected sensing data and the cumulative coverage of the sensing task requirements. In our simulation, the vehicles continuously collect sensing data if there are sensing task requirements, and the temporal coverage of the collected sensing data may exceed the required range of the sensing task. Therefore, the coverage of the collected sensing data may be larger than the cumulative requirements of the sensing tasks.

**Figure 8:**
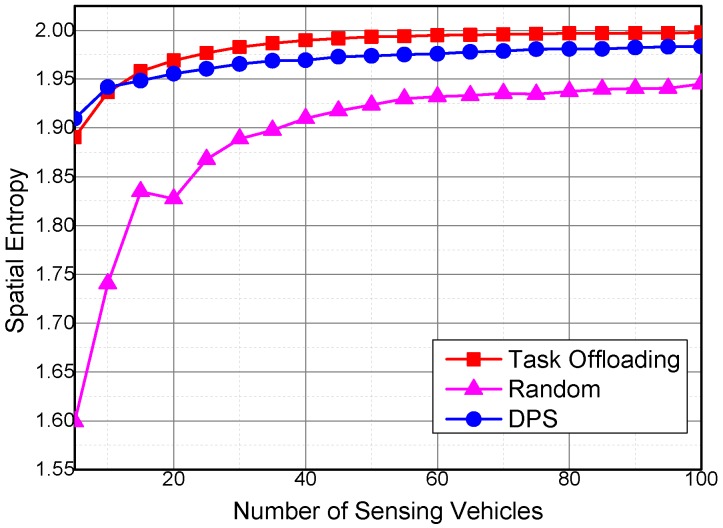
Spatial entropy with different numbers of sensing vehicles.

Figure 8 shows the spatial entropy with different numbers of sensing vehicles. Spatial entropy is able to reflect the degree of uniformity of the collected sensing data. The spatial entropy of the collected sensing data is large when the sensing data spatial distribution degree is high. The spatial entropy of the three algorithms increase with the increase in the number of sensing vehicles, which means that more sensing vehicles can collect more uniformly distributed sensing data. Given that the Random algorithm chooses the sensing vehicles randomly at the beginning of the simulation, the repetition rate of the collected sensing data in the Random algorithm is higher than that of the other two algorithms. As a result, the spatial entropy of Random is smaller than Task Offloading and DPS under the same number of sensing vehicles.

By offloading the tasks between the sensing vehicles, the Task Offloading algorithm executes the sensing tasks by the vehicles with high utilities. DPS selects the vehicles with higher utilities at the beginning of the simulation. Thus, both Task Offloading and DPS are able to improve the spatial distribution of the collected sensing data. As shown in Figure 8, the Task Offloading algorithm has bigger spatial entropies than the DPS algorithm, because the Task Offloading algorithm further optimizes the task execution efficiency by decomposing and offloading the sensing tasks to the vehicles with higher utilities. However, when the number of selected vehicles is too small (i.e., <10), the chance for the sensing vehicles to collaborate with each other is too small. Thus, the spatial entropy of the Task Offloading algorithm becomes lower than that of the DPS algorithm.

**Figure 9:**
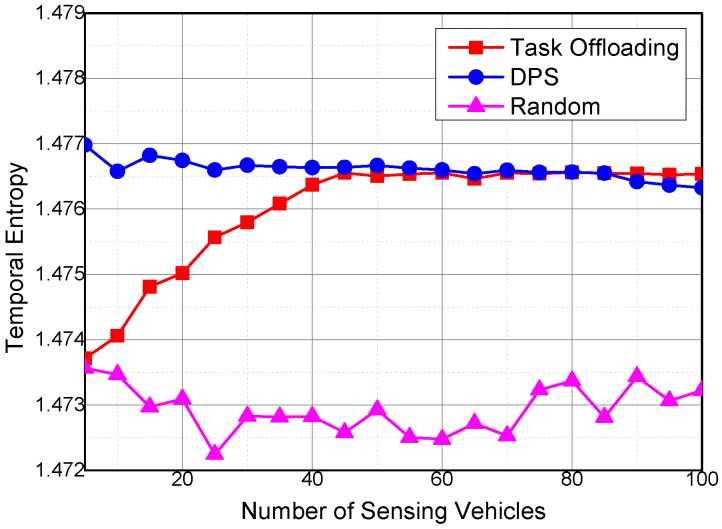
Temporal entropy with different numbers of sensing vehicles.

Temporal entropy reveals the stability of the sensing system on collecting the sensing data continuously. As shown in Figure 9, the temporal entropies of the collected sensing data by the three algorithms show different variation trends with the increase in number of sensing vehicles. The temporal entropy of Task Offloading increases with the increase in number of vehicles collecting the sensing data. The growth rate of the temporal entropy of the Task Offloading algorithm decreased with the increase in number of sensing vehicles. The temporal entropy of DPS slightly decreases along with the increase in number of sensing vehicles. The temporal entropies of Task Offloading and DPS are both higher than that of Random, which means that Task Offloading and DPS are better at continuously collecting sensing data.

The temporal entropy of the Random algorithm fluctuates around a small value. The Random algorithm selects sensing vehicles randomly from the candidates; as a result, the Random algorithm experiences difficulties in collecting sensing data continuously. For the same reason, the fluctuation degree of the Random algorithm is also higher than that of the other two algorithms. When the number of sensing vehicles is small, the temporal entropy of Task Offloading is lower than DPS, given that DPS selects vehicles according to their utilities, whereas Task Offloading distributes sensing task randomly to the sensing vehicles at the beginning of the simulation. When the number of sensing vehicles is small, there are less chances of offloading sensing tasks among sensing vehicles. Therefore, the temporal entropy of the Task Offloading algorithm is smaller than that of DPS and higher than that of Random. More opportunities to offload sensing tasks among sensing vehicles are generated with the increase in number of sensing vehicles. Thus, the temporal entropy of Task Offloading exceeds that of DPS at high number of sensing vehicles.

#### 5.1.2. Mean Value of Task Durations

The performance of algorithms under different temporal coverage expectations of the sensing tasks is evaluated. The task coverage ratio is the ratio between the cumulative tempo-spatial coverage of the collected sensing data and the cumulative tempo-spatial coverage of the sensing tasks.

**Figure 10:**
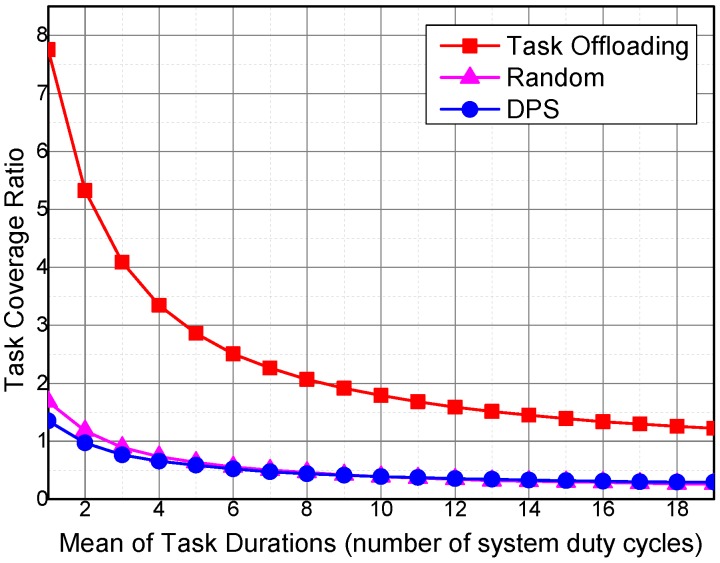
Impact of task durations on task coverage ratio.

Figure 10 shows the impact of the task duration for he required sensing data temporal coverage on task coverage ratio. The task coverage ratio decreases with the increase in duration of the generated sensing tasks. The increase in temporal coverage duration of a single task would increase the cumulative task duration for the required sensing data temporal coverage, thereby decreasing the task coverage.

In our simulations, each vehicle collects sensing data according to the tempo-spatial coverage requirements of the sensing tasks. Therefore, more sensing task tempo-spatial coverage requirements lead to more sensing data collected by the vehicles. However, the increase rate of the amount of collected sensing data is slower than that of the required tempo-spatial coverage of a task. As a result, the task coverage ratio exponentially decreases with the increase in sensing task duration for the required temporal coverage. The task coverage ratio of Task Offloading is higher than that of the other two algorithms, because sensing tasks are executed by the sensing vehicles with higher sensing utilities.

The sensing data coverage ratio of Task Offloading is much higher than that of DPS and Random, because Task Offloading considers the lifetime of the collected sensing data, whereas the collected sensing data in Random and DPS cover only one system duty cycle. Furthermore, Task Offloading is able to offload sensing tasks to sensing vehicles with the highest utilities for a specific sensing task, and the sensing data collected by the task performer offer the highest coverage ratio.

**Figure 11:**
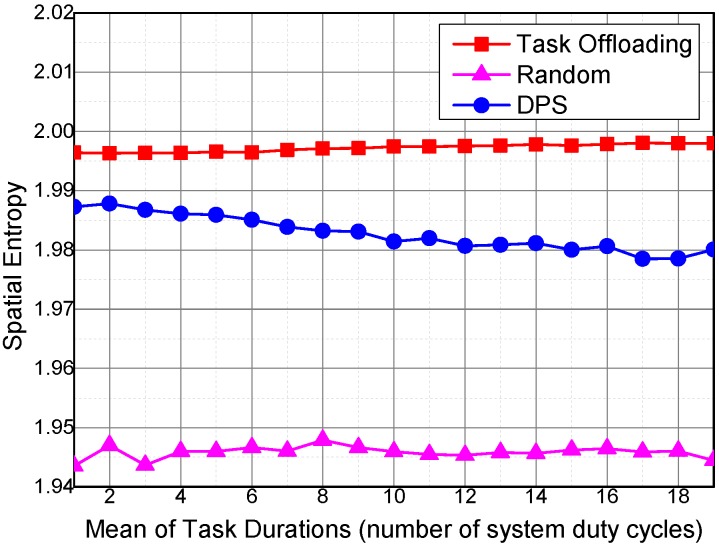
Spatial entropy under different mean values of task durations.

Figure 11 reveals the variations of spatial entropy under different sensing task requirements on the temporal coverage duration of the collected sensing data. The required temporal coverage duration of a sensing task has little influence on the spatial entropy of the three algorithms. The spatial entropy of DPS decreases slightly with the increase of sensing task requirements of the temporal coverage duration. In particular, the spatial entropies of Task Offloading and Random are barely affected by the sensing task requirements. The Random algorithm has the lowest spatial entropy, because vehicles are selected randomly and the overlapping ratio of the collected sensing data is high. The DPS and Task Offloading algorithms select sensing vehicles based on their utilities, and thus the collected sensing data are uniformly distributed. As shown in Figure 11, the Task Offloading is able to obtain higher spatial uniformity than DPS in terms of task offloading among sensing vehicles.

**Figure 12:**
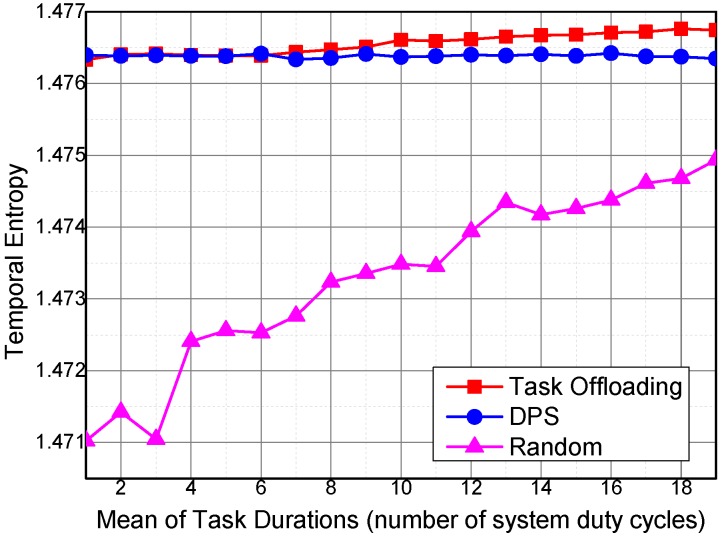
Temporal entropy under different mean values of task durations.

Figure 12 shows the variation trends of temporal entropy of the three algorithms under different sensing task requirements on the temporal coverage duration. The temporal entropy of Task Offloading and DPS is barely affected by the required temporal coverage duration of a sensing task. The temporal entropy of the Random algorithm increases with the increase in required temporal coverage duration of the collected sensing data for a sensing task. The required temporal coverage duration of a sensing task affects the amount of collected sensing data; more sensing data entail more requirements on the temporal coverage of the collected sensing data. The Task Offloading algorithm optimizes the execution of sensing task by decomposing and offloading the sensing tasks to the sensing vehicles with higher utilities to collect sensing data with less temporal overlapping ratio. Therefore, the temporal entropy of Task Offloading is higher than that of DPS and Random.

#### 5.1.3. Impact of Task Generation Rate

**Figure 13:**
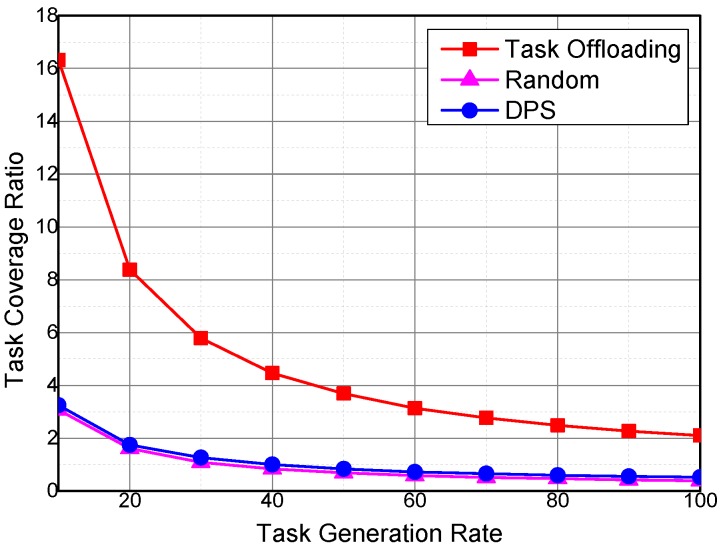
Impact of task generation rate on task coverage ratio.

The impact of task generation rate on the sensing task coverage ratio and tempo-spatial entropy of the collected sensing data is evaluated. The task generation rate denotes the amount of generated sensing task in a system duty cycle. Figure 13 shows the impact of the sensing task generation rate on the task coverage ratio. The task coverage ratio of the three algorithms decreases with the increase in sensing task generation rate. Although generating more sensing tasks can increase the requirements on the tempo-spatial coverage of collected sensing data, and then more sensing data will be collected by the sensing vehicles, the increase rate of the cumulative coverage of the collected sensing data is slower than the cumulative required tempo-spatial coverage duration of sensing data. Thus, the sensing task coverage ratio decreases with the increase in sensing task generation rate.

The task coverage ratio of Task Offloading is higher than that of DPS and Random because the sensing tasks are offloaded to the sensing vehicles with higher utilities. The impact of task generation rate on the task coverage ratio is similar to the impact of required temporal coverage duration of the sensing data for a sensing task on the task coverage ratio, because sensing task generation rate is increased to increase the tempo-spatial coverage requirements of the sensing tasks.

**Figure 14:**
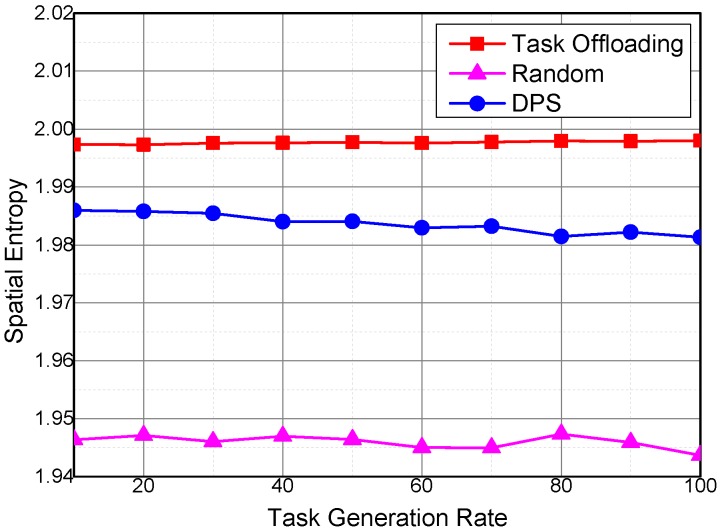
Spatial entropy under different task generation rates.

**Figure 15:**
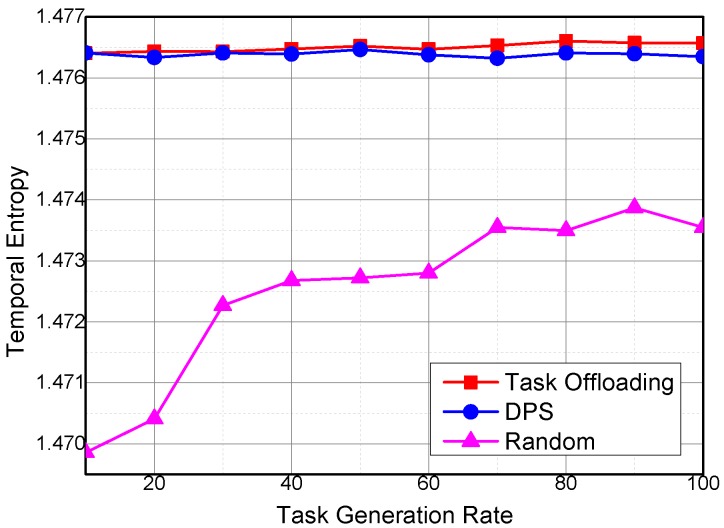
Temporal entropy under different task generation rates.

For the same reason, spatial and temporal entropies have similar variation trends under different task generation rates as that under different temporal coverage of sensing tasks, as shown in Figure 14 and Figure 15.

## 6. Conclusions

Task Offloading algorithm is proposed for vehicular sensing systems to collect comprehensive covered sensing data. In the proposed Task Offloading algorithm, the mobility of the vehicles is modeled with a time-continuous Markov chain, and the utility function of a vehicle to a sensing task is established according to the required tempo-spatial coverage of the sensing data, required sensing data types, sensing interfaces of the vehicle, and mobility trace of the sensing vehicle. The sensing tasks are decomposed and offloaded to other participants according to the utilities of the sensing vehicles. Real trace-driven simulations show that the proposed Task Offloading algorithm is able to obtain considerably more uniformly-distributed tempo-spatial sensing data and higher task coverage ratio than the other algorithms, namely, Random and DPS. 

## Figures and Tables

**Table 1:** List of notations.

Notation	Description
*I*	The set of subareas
i,j∈I,1⩽i⩽N	One subarea
$w_{l}$	Life cycle of the *l*th type of sensing data
α,β	Sensing vehicles
$Y_{\alpha }=\left(y_{1}^{\alpha },y_{2}^{\alpha },\cdots ,y_{M}^{\alpha }\right)$	Sensing interfaces of vehicle *α*
$\xi_{i}^{\alpha }$	The time that vehicle *α* arrives at subarea *i*
$\theta_{i}$	Residence time of vehicle in subarea *i*
$Z\left(t\right)$	Valid sensing data in the sensing data center at time *t*
$\zeta_{i,l}\left(t\right)$	Valid function of the sensing data of type *l* in subarea *i* at time *t*
$X=\left\{X\left(t\right),t⩾0\right\}$	Mobility procedure of a vehicle
$P_{ij}\left(t\right)$	The transition probability of a vehicle from sub area *i* to sub area *j* after time duration *t*
$T_{k}$	Sensing task *k*
$u_{\alpha }\left(T_{k}\right)$	Utility of sensing vehicle *α* to task $T_{k}$

**Table 2:** Simulation parameters.

Parameter	Default Value
Simulation time	533,315 s
Number of participant vehicles	60
System duty cycle	1000 s
Sensor types	10
Sensing data life cycle	10 system duty cycles
Area partition	100 (10×10)

## Abbreviations

The following abbreviations are used in this manuscript:

GPS	Global Position System
MOSDEN	MObile Sensor Data ENgine
DPS	Dynamic Participant Selection
